# Mistaken perception of lipid intake and its effects: a randomized trial

**DOI:** 10.1186/s40795-017-0193-8

**Published:** 2017-09-19

**Authors:** Mariana Carvalho de Menezes, Sueli Aparecida Mingoti, Raquel de Deus Mendonça, Aline Cristine Souza Lopes

**Affiliations:** 1 Department of Nutrition, Research Group in Nutrition Interventions, University of Minas Gerais, 190 Alfredo Balena ave. Escola de Enfermagem, Santa Efigênia, Belo Horizonte, MG 30190-100 Brazil; 2 Department of Statistics, University of Minas Gerais, Antônio Carlos ave. 6627 Campus Pampulha, Belo Horizonte, MG 30161-970 Brazil

**Keywords:** Intervention studies, Transtheoretical model, Stages of change, Feeding behavior, Eating

## Abstract

**Background:**

Although the Transtheoretical Model (TTM) is promising for behavioral interventions, it may be limited by an inability to understand perceptions of food consumption. The following questions and gaps presented by the scientific community prompted this study: What is the concordance between perceived and actual food consumption? What proportions of individuals are in the pseudo-maintenance (PM) stage (overly optimistic perception of fat consumption)? What is the proportion of individuals in the non-reflective action stages (adequate fat intake but do not recognize it)? Is it necessary to develop specific strategies for individuals in these stages? Therefore, the present study aimed to identify the proportion of “pseudo-respondents,” or those in the PM and non-reflective action stages, and to explore subgroup effects by PM classification.

**Methods:**

In a previously conducted randomized controlled trial, participants in the usual care group (UCG) and the TTM-intervention group (TM-IG) were post-hoc classified as “true respondents” or “pseudo-respondents”; the latter included those in the PM (mistakenly perceived their lipid intake as adequate) or non-reflective action (did not recognize the adequacy of their lipid intake) stage. The 6-month TTM-based intervention for fat consumption was performed with a sample of Public Health Service users.

**Results:**

Seventy-one women completed all of the phases. About half of the participants were in the PM stage (UCG: 14 of 31; TM-IG: 19 of 40), and only two were in the non-reflective action stage. Post-intervention, PM individuals in the TM-IG evolved differently, with greater progression to later stages of change and reduced calorie intake, weight, and body mass index (*p* < 0.05).

**Conclusions:**

Owing to the high proportion of participants in the PM stage and the differing performance, this stage is important. The intervention had a previously unreported differential effect on the progression of the stage of change and nutritional status by PM classification.

**Trial registration:**

RBR-5TDHZY (retrospectively registered in August 2017 in Brazilian Registry of Clinical Trials).

**Electronic supplementary material:**

The online version of this article (10.1186/s40795-017-0193-8) contains supplementary material, which is available to authorized users.

## Background

Given the current health situation characterized by a remarkable prevalence of inadequate eating practices, the Transtheoretical Model (TTM) stands out as one of the most promising theoretical models for understanding and predicting behavior change [1]. The TTM features a core concept used to delineate more specific and adapted nutritional interventions termed the stages of change (SOC), which refer to when the behavioral change occurs and represent the different degrees of individuals’ motivation to modify their health behaviors. The stages are well described and include precontemplation, contemplation, preparation, action, and maintenance [2]. However, there are limitations and methodological challenges involving the measurement of the SOC and hence developing appropriate interventions [3].

The SOC of individuals is classified by applying an algorithm that refers to a specific questionnaire for one or more food items (e.g., fat consumption), which may be based on the perception of food and/or the assessment of food consumption [4]. Regarding the algorithm that is based solely on food perception, it is important to consider that individuals usually have an extremely optimistic view of their consumption, especially fat consumption [5]. Consequently, the misperception of food intake can lead to misclassification of the SOC, hindering interventions.

Accordingly, in 1996, Steptoe et al. [6] proposed reclassifying individuals classified in the maintenance stage who had a perception of adequate fat intake but who actually had high fat consumption into the “pseudo-maintenance (PM)” stage. Similarly, in 2003, Ma et al. [7] proposed reclassifying individuals who perceive their fruit and vegetable intake is inadequate despite having adequate consumption in the “non-reflective action” stage. It is unknown whether specific intervention strategies need to be developed for individuals in these new SOC [7].

As a substantial percentage of individuals usually misperceive their eating behavior, it is challenging to define the stages of behavioral change. Classification errors are reportedly persistent and can reduce the effectiveness of interventions [5]. Nevertheless, interventions based on the TTM usually do not investigate the concordance between perceived and actual food intake.

The following questions and knowledge gaps presented by the scientific community prompted this study: considering eating practices related to fats, what is the concordance between perceived and actual food consumption? What is the proportion of individuals in the PM stage, i.e., those who have an overly optimistic perception of their fat consumption? What is the proportion of individuals in the non-reflective action stage, i.e., those who have adequate fat intake but do not recognize it? Is it necessary to develop specific strategies for individuals reclassified in these stages? Therefore, the present study aimed to identify the proportion of individuals classified in the PM and non-reflective action stages for fat consumption as well as to explore subgroup effects by PM classification of a previously conducted, effective intervention. The main aim of the TTM-based intervention was reduction in the consumption of high-fat foods [8, 9].

The hypotheses were the following: (1) the proportion of individuals in the PM stage is high, (2) the proportion of individuals in the non-reflective action stage is low, and (3) individuals in the PM stage are less sensitive to interventions.

## Methods

### Study design and participants

In a previously conducted randomized controlled trial, participants in the usual care group (UCG) and the Transtheoretical Model-intervention group (TM-IG) were *post-hoc* classified as true or pseudo-respondents. Details of the intervention have been published elsewhere [8, 9].

The intervention, which aimed to reduce fat consumption, was developed as part of a public health promotion service called the *Programa Academia da Saúde*, which provides users access to regular physical exercise and nutritional counseling. This service is located in Belo Horizonte, which is the sixth most populous city in Brazil, with 2,375,151 inhabitants [10].

This study involved a sample of participants from the public service aged ≥20 years. The eligibility criteria were as follows: (1) no previous participation in any nutritional intervention that addressed fat consumption, (2) regular use of the service, and (3) written informed consent to participate. The Institutional Review Boards of the University (ETIC 103/07) and the Municipality approved the study protocol (087/2007).

A random sample was used to ensure representativeness. The study population comprised 336 participants. The sample size was determined by a normal bilateral *Z*-test to compare the proportions of two independent groups, with 80% power to detect a 15% difference between groups at a 5% significance level and assuming 53% attrition. Thus, 168 individuals were selected for the first study phase in 2009, which aimed to validate the algorithm proposed by Greene & Rossi [11]. The algorithm validation phase has been previously described in detail [12]. Briefly, during validation, the algorithm for fat intake and three 24-h dietary recalls (24DRs) were administered every other day, including one weekend day. Initially, the individuals were classified according to the algorithm; then, they were reclassified according to mean lipid intake based on the three 24DRs.

In the second study phase involving the TTM-based intervention in 2009, 118 individuals of the original 168 individuals participated (lost to follow-up: declined, insufficient data, or health or family problems). The participants were simple randomized into the UCG and TM-IG using a random number table prepared by a professor with no involvement in the intervention; the researchers were blinded to the allocations. After postgraduate students obtained the participant’s consent, the appropriate numbered sealed envelope was opened. Among the 118 participants, 97 completed the baseline questionnaire and started the intervention. Additional details about the sample calculation and attrition have been reported previously [8].

### Measures

Analyzed data included sociodemographic characteristics, self-reported diseases, food consumption, SOC, and measured anthropometric parameters. The questionnaire was previously validated with service users and was determined to be culturally appropriate for the study population [8]. The questionnaire included questions about sociodemographic variables (i.e., sex, age, educational level [years], occupation status, number of household members, and household per capita income), and self-reported non-communicable diseases. Food consumption was assessed using the three 24DRs and questions about eating habits related to fat.

Anthropometric measurements included weight, height, waist circumference, and hip circumference using techniques recommended by the World Health Organization (WHO) [13, 14]. The measurements were obtained in the morning with the individual fasting, barefoot, and wearing lightweight clothes. Periodically trained dietitians collected all data through face-to-face interviews.

Body mass index (BMI) was calculated from weight and height and classified according to the criteria recommended by the WHO [13] for adults and the Nutrition Screening Initiative [15] for elderly participants. The WHO criteria [14] were used to evaluate waist circumference and the waist/hip ratio. Both circumferences were measured three times, and the means were used for analysis.

Food consumption was investigated as follows: (1) the average of three 24DRs administered on alternate days including a weekend day and (2) direct questions related to dietary lipid intake, such as methods of food preparation, including removal of visible fat from red meat and skin from chicken; types of vegetable oil and milk consumed; and frequencies of consumption of fish and high-fat foods ([never/almost never, <2 times/week, 2–3 times/week, 4–5 times/week, or every day]) [12].

Dietwin software was used to analyze food consumption from the 24DRs. The software was supplemented with a food composition table subsidized by the Ministry of Health of Brazil that was constructed from a laboratory analysis of food chemistry composition and complemented by two other tables used in Brazil [16, 17].

The qualitative adequacy of nutrient intake was assessed using the Institute of Medicine Dietary Reference Intakes.

The SOC for fat consumption was classified using the algorithm proposed by Greene & Rossi [11], which has been validated in Brazil [12]. According to this instrument, the SOC is first classified by evaluating an individual’s perception of lipid intake (“*I need your honest opinion about your change of fat intake. Do you consistently avoid eating high-fat foods?*”). Second, mean lipid intake values from the three 24DRs were evaluated for the individuals classified into the action and maintenance stages based on self-perceived behavior.

**Fig. 1:**
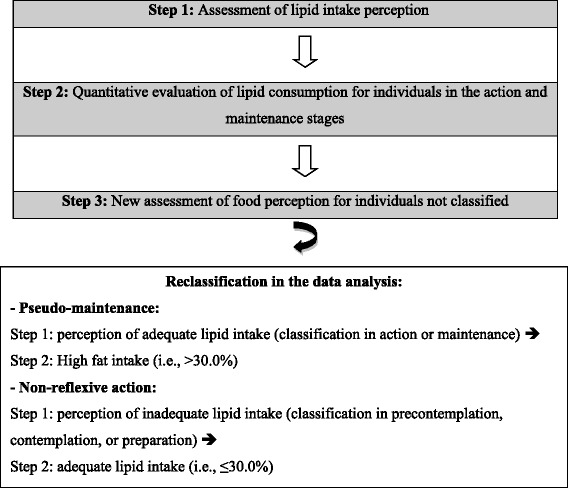
Steps for classification and reclassification of stages of change for fat consumption

Third, individuals whose self-perceived behavior was not confirmed (i.e., failed to meet the behavioral criterion of fat intake ≤30%) were reclassified through a new self-assessment of their intention to change their eating behavior (Questions: “Do you almost always take the skin off your chicken?”; “Do you often eat reduced-fat or low-fat cheese?”; “You often use light, fat-free, or no salad dressing?”; “Do you sometimes eat fruits as vegetables in snacks?”; “Do you often eat bread, rolls, or muffins without butter or margarine?”) (Fig. 1). Interventions tailored to individuals’ readiness to change were subsequently developed.

To explore the subgroup effects of the previously conducted intervention, secondary analyses of the data were performed. The primary variable was created through *post-hoc* classification of participants into the PM [6] and non-reflective action [7] stages. Participants were classified into the PM stage if they perceived their lipid intake as adequate, i.e., those classified in the action or maintenance stage in the first step of the algorithm but had an elevated fat intake according to the 24DR food consumption assessment in the 2-step algorithm. The non-reflective action stage included individuals who reported having inadequate fat intake, i.e., those classified in the early stages (i.e., precontemplation, contemplation, and preparation) in the first step despite showing adequate fat consumption (i.e., ≤ 30.0%) according to the 24DR assessment in the 2-step algorithm (Fig. 1).

### Intervention

The UCG performed the routine activities of the *Programa Academia da Saúde*, including aerobic and anaerobic exercise (180 min weekly) and open group education regarding nutrition. The group education sessions occurred monthly and involved topics related to health promotion, prevention, and the control of chronic diseases but not fat consumption.

In addition to these routine activities, the TM-IG underwent TTM-based interventions tailored to the subgroups formed from the SOC classification of individuals for fat consumption: pre-action group and action group [9].

Interventions were developed by a multidisciplinary team that included nutritionists and psychologists theoretically aligned with the TTM. The group technique was concordant with the autonomy principle [8].

The nutritional and health needs of the participants and the four pillars of TTM (i.e., stages and processes of change, self-efficacy, and decisional balance) were considered when developing the themes of the interventions. The interventions aimed to increase participants’ confidence (self-efficacy), raise awareness of the benefits of healthy lipid intake, and reduce the barriers to behavioral changes (decisional balance) [8, 9].

In the pre-action group, the use of cognitive processes of change was predominant, whereas the use of behavioral processes was predominant in the action group. The pre-action intervention aimed to facilitate decision-making through the appreciation of participants’ experiences as well as experienced barriers to changing eating habits. The interventions for the action group emphasized culinary workshops, which allowed individuals to experience daily recipes and the degustation of various dishes. Further details of the intervention are described by Siqueira [9].

The intervention comprised 20 workshops over six months: 10 each for the pre-action and action groups. A total of 54 workshops were held. Additional details about the routine activities and TTM intervention have been reported previously [8].

### Statistical analysis

Descriptive statistical analysis was performed. The Shapiro-Wilk test was initially applied to assess the normality of quantitative data. Normally and non-normally distributed variables are presented as mean ± standard deviation and median and interquartile range (i.e., first quartile, third quartile), respectively. The *χ* ^2^ test, Fisher’s exact test, Student’s *t*-test for independent samples, and Mann–Whitney *U*-test were used for intergroup comparisons. Meanwhile, the McNemar test, paired Student’s *t*-test, and Wilcoxon signed rank test were used for intragroup comparisons. The level of significance for all tests was 5%.

Data were analyzed with SPSS statistical software (version 16.0, SPSS Inc., Chicago, IL, USA). Prior to the analysis, the consistency of the database was examined by checking that the data recorded in the questionnaire and entered into the database were concordant.

## Results

Of the 97 individuals who started the intervention, 79% completed the study (*n* = 77). Individuals who attended less than 50% of all workshops were excluded from the analysis [18]. In addition, male participants (*n* = 6) were excluded from the analysis in order to obtain a homogeneous sample. Thus, 71 women remained in the final analysis. Attrition data, comparisons of individuals who dropped out with those who completed the study, and intervention effectiveness have been reported previously [8].

The participants had a mean age of 57.9 ± 11.7 years and were characterized by low education and per capita income (Table 1), a high prevalence of overweight (46 of 71 participants), and a high prevalence of chronic diseases such as high blood pressure (42 of 71) and hypercholesterolemia (27 of 71). Nevertheless, most individuals were classified into the action stages (UCG: 17 of 31; TM-IG: 20 of 40), which included the action (UCG: 10; TM-IG: 11) and maintenance (UCG: 7; TM-IG: 9) stages. The others were classified into the pre-action stages (UCG: 14 of 31; TM-IG: 20 of 40), which included the precontemplation (UCG: 3; TM-IG: 2), contemplation (UCG: 4; TM-IG: 7), and preparation (UCG: 7; TM-IG: 11) stages.

**Table 1:** Baseline sociodemographic characteristics according to pseudo-maintenance classification

Sociodemographic characteristics	Usual care group (*n* = 31)			Transtheoretical Model-intervention group (*n* = 40)		
	PM^a^ – no (*n* = 17)	PM^a^ – yes (*n* = 14)	*p* value	PM^a^ – no (*n* = 21)	PM^a^ - yes (*n* = 19)	*p* value
Age (years)	57.5 ± 16.1	63.9 ± 9.4	0.205^b^	56.0 ± 9.8	55.8 ± 9.8	0.946^b^
Family income per capita ($)	224.5 (128.6–340.6)	132.8 (77.1–242.6)	0.164^c^	300.0 (144.9–273.6)	187.1 (149.7–362.2)	0.130^c^
Residents per household	3.0 (2.0–4.0)	3.5 (2.0–4.3)	1.000^c^	3.0 (2.5–4.5)	3.0 (3.0–5.0)	0.778^c^
Education (years)	4.0 (1.5–10.0)	3.5 (1.8–8.0)	0.356^c^	4.0 (3.5–8.0)	7.0 (4.0–11.0)	0.145^c^
Occupancy (%)			0.709^d^			0.105^d^
With fixed income	70.6% (*n* = 12)	64.3% (*n* = 9)		42.9% (*n* = 9)	68.4% (*n* = 13)	
Without fixed income	29.4% (*n* = 5)	35.7% (*n* = 5)		57.1% (*n* = 12)	31.6% (*n* = 6)	

^a^PM, pseudo-maintenance; ^b^Student’s *t-*test; ^c^Mann–Whitney *U-*test; ^d^Pearson *χ* ^2^ test. Normally and not normally distributed variables are expressed as mean ± standard deviation and median (interquartile range; first to third quartile), respectively

**Fig. 2:**
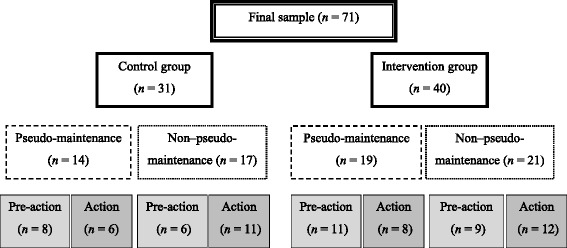
Classification of pseudo-maintenance and subject allocation to the pre-action and action intervention subgroups

Regarding the post-hoc reclassification of these participants, approximately half were in the PM stage. Figure 2 presents the classification of PM and participant allocation to the pre-action and action intervention subgroups. Regarding the non-reflective action stages, only one individual each in the UCG and TM-IG was reclassified; both individuals were classified into the preparation stage according to their perception of lipid intake although they had adequate fat consumption. Given the number of individuals classified in the PM stage, the effect of the intervention with respect to the classification at this stage was analyzed.

At baseline, participants classified into the PM stage had similar sociodemographic characteristics to those not classified into the PM stage (Table 1).

PM participants in the TM-IG evolved distinctly from the other participants, with greater progression to the later stages of behavioral change (Table 2) and reductions in calorie intake (pre-intervention: 1983.6 ± 557.0 kcal; post-intervention: 1685.6 ± 354.0 kcal; *p* = 0.001), weight, and BMI (Table 3). In addition, these participants exhibited a decreased frequency of high-fat food consumption (i.e., fried foods, fried snacks, burgers, and sausages [from 36.8% to 10.5%; *p* = 0.063]) and a significant improvement in their body perception; after the intervention, they perceived themselves as less “fat” (from 15 of 19 to 12 of 19 participants) and more “normal” (from 3 of 19 to 6 of 19 participants), although the difference was not statistically significant (*p* = 0.083).

**Table 2:** Pre- and post-intervention changes in the stages of change for fat consumption according to pseudo-maintenance classification

Movement in stages	PM^a^ – no		PM^a^ – yes		*p* value
	*n*	Percent	*n*	Percent	
Usual care group					
Progression^b^	7	43.8	9	64.3	0.516^c^
No change^d^	3	18.7	2	14.3	
Regression^e^	6	37.5	3	21.4	
Transtheoretical Model-intervention group					
Progression^b^	4	19.0	11	57.9	0.031^c^
No change^d^	10	47.6	6	31.6	
Regression^e^	7	33.3	2	10.5	

^a^ *PM* pseudo-maintenance; ^b^Progression: participants at baseline who progressed to “upper” stages of change at post-intervention; ^c^Pearson *χ* ^2^ test; ^d^No change: participants at baseline who were in the same stage of change post-intervention; ^e^Regression: participants at baseline who regressed to “lower” stages of change post-intervention

**Table 3:** Pre- and post-intervention differences in anthropometric measurements according to pseudo-maintenance classification

Usual care group (*n* = 31)						
Anthropometric characteristics	Non–pseudo-maintenance group (*n* = 17)			Pseudo-maintenance group (*n* = 14)		
	Pre-intervention	Post-intervention	*p* value	Pre-intervention	Post-intervention	*p* value
Weight (kg)	64.5 ± 11.2	64.4 ± 11.5	0.888^a^	67.4 ± 16.0	68.3 ± 17.8	0.071^a^
BMI^b^ (kg/m^2^)	27.2 ± 5.2	27.2 ± 5.2	0.993^a^	28.3 ± 5.9	28.6 ± 6.4	0.271^a^
WC^c^ (cm)	83.2 ± 9.0	83.9 ± 8.0	0.217^a^	88.0 ± 12.0	88.5 ± 11.7	0.677^a^
WHR^d^	0.819 ± 0.06	0.820 ± 0.06	0.902^a^	0.839 ± 0.06	0.847 ± 0.06	0.627^a^
Transtheoretical Model-intervention group (*n* = 40)						
Anthropometric characteristics	Non–pseudo-maintenance group (*n* = 21)			Pseudo-maintenance group (*n* = 19)		
	Pre-intervention	Post-intervention	*p* value	Pre-intervention	Post-intervention	*p* value
Weight (kg)	68.1 ± 11.1	67.3 ± 11.2	0.194^a^	68.6 ± 12.1	67.4 ± 11.8	0.048^a^
BMI^b^ (kg/m^2^)	28.5 ± 3.8	28.1 ± 3.9	0.155^a^	27.6 ± 4.2	27.1 ± 4.2	0.028^a^
WC^c^ (cm)	85.0 ± 9.3	84.8 ± 7.3	0.873^a^	84.9 ± 10.0	83.6 ± 8.1	0.193^a^
WHR^d^	0.824 ± 0.05	0.816 ± 0.04	0.437^a^	0.834 ± 0.07	0.819 ± 0.06	0.106^a^

^a^Paired Student’s *t*-test. Normally distributed variables are expressed as mean ± standard deviation. ^b^ *BMI* body mass index; ^c^ *WC* waist circumference; ^d^ *WHR* waist/hip ratio

Participants in the PM stage who were reclassified into the action group had decreased caloric intake (pre-intervention: 2265.7 kcal [1504.5–2846.1]; post-intervention: 1679.7 kcal [1253.6–2073.1]), weight (pre-intervention: 71.3 ± 13.7 kg; post-intervention: 70.0 ± 13.3 kg), and BMI (pre-intervention: 28.5 ± 5.4 kg/m^2^; post-intervention: 28.0 ± 5.2 kg/m^2^).

Participants in the UCG who were not classified into the PM stage exhibited significantly increased lipid consumption (pre-intervention: 27.2 [25.3–30.8] %kcal; post-intervention: 33.1 [29.5–35.7] %kcal; *p* = 0.015), including saturated fatty acids (pre-intervention: 7.8 [6.7–8.4] %kcal; post-intervention: 9.3 [7.0–12.1] %kcal; *p* = 0.034). There were no statistically significant differences in lipid consumption for the TM-IG non-PM and UCG PM groups and in body perception for the other groups.

## Discussion

Starting from a baseline sample composed mostly of women in the action and maintenance stages, the post-hoc reclassification revealed a very small proportion of individuals in the non-reflective action stage and a large proportion in the PM stage. After participating in the TTM-based interventions, individuals in the PM stage evolved differently from others, exhibiting results that were more satisfactory than what was expected.

Regarding the non-reflective action stages, very few individuals did not recognize that they had adequate fat consumption. In contrast, Toral & Slater [4] found greater percentages of individuals in non-reflective action regarding fat consumption. However, their study involved teenagers, and the non-reflective action stage was originally developed for fruit and vegetable consumption [7]. Therefore, further investigations are required to confirm if the non-reflective action stage is not relevant for fat consumption.

On the other hand, approximately half of all participants were classified into the PM stage. This may be because most of the women were classified in the action or maintenance stage at baseline despite poor eating habits and the high prevalence of overweight and diseases [19–21].

Although studies do not usually evaluate the proportion of individuals in PM, the present results, specifically the large proportion of individuals in the later stages of the TTM at baseline, are corroborated by other investigations [20, 21]. Given the small sample size for these results, other studies should be conducted to explore whether the baseline classification accurately reflects actual eating perception and motivation to change.

Misconceptions regarding the perception of fat intake adequacy and consequently the design of interventions if individuals are not reclassified have been investigated previously [5, 22]. Misclassification based on perception can be due to an individual’s difficulty estimating fat consumption [23], which arises from difficulty visually assessing the presence of fat in food, because fat is usually added as an ingredient [4].

In addition, Plotnikoff et al. [5] suggest individuals may have exaggerated beliefs regarding their actions to reduce fat intake that arise from cognitive biases activated when analyzing this behavior. Making one or two changes such as reducing the consumption of fried snacks for example, may be sufficient to create an impression of having adequate fat consumption. Accordingly, the only question of intention used in the algorithm (i.e., “do you consistently avoid eating high-fat foods?”) may favor individuals who remember such changes in their eating behavior, resulting in a consideration of adequate fat intake in their diet.

The high percentage of individuals classified in the PM stage in the present study is corroborated by other studies evaluating this stage [4, 23]. However, other studies that evaluated the behavior of individuals in PM after participating in an intervention were not found. In the present study, women in PM exhibited better improvements in anthropometric parameters and food consumption as well as SOC progression.

To better understand the results exhibited by the women in PM in the TM-IG, it is important to consider that they participated in different intervention workshops (i.e., pre-action and action groups; Fig. 2).

Given the small group sizes, descriptive and exploratory statistics were used, and all participants in the TM-IG exhibited reduced calorie consumption, weight, and BMI (see Additional file 1) independent of the subgroups, i.e., non-PM in pre-action or action and PM in pre-action or action. However, the degree of improvement differed. Thus, this study is exploratory and pioneering, by comparing these groups of participants. Furthermore, the initial analyses are essential for formulating additional hypotheses with greater precision to guide future studies.

Interventions were more effective for women in PM reclassified into the action group; their median caloric intake decreased 503.3 kcal after the intervention, with post-intervention differences in mean weight (−1.26 kg) and BMI (−0.55 kg/m^2^). It is possible that these women reported healthy eating behaviors because they had already initiated healthy changes [7] but were still incipient to significantly reflect on the adequacy of their lipid intake. As a result, this classification into the PM stage would have occurred not because of a poor perception of their diet but rather because they have not achieved the recommended values for lipid intake (i.e., ≤30%) [4].

In addition, individuals with high consumption of lipids need to change multiple dietary habits, such as methods of food preparation, avoiding high-fat foods, and changing the type and amount of vegetable oil used. All these factors (high fat intake at baseline, misperception of their own consumption of lipids, and difficulty in changing dietary habits) may have influenced the evolution through the stages. Thus, the proposed intervention for the action group, which aimed to facilitate nutritional knowledge and suggested strategies for action, may have encouraged new attitudes among participants who were already in the process of change.

In contrast, women in the PM stage who were allocated to the pre-action group only initially believed they had appropriate lipid intake (i.e., step 1 of the algorithm). However, when asked about their food perception again, they revised their perception and considered themselves in the pre-action stage (i.e., step 3 of the algorithm). These women, who previously believed they had adequate fat intake, may have felt more motivated to change when they became aware of their true behavior. This subsequently prompted better evolution through the stages than the women not classified in the PM stage.

It is worth noting that, although some authors report the need to develop specific strategies for individuals in the PM stage [7], the present study is pioneering in the analysis of participants in the PM stage after an intervention. Furthermore, the participants exhibited positive changes even though the intervention was not developed specifically for their group. These preliminary results indicate that allocation of individuals in the PM stage to workshops for pre-action or action groups seems appropriate, showing the importance of valuing food perception in the design of TTM-based interventions [23]. These novel hypotheses must be investigated by further studies with larger samples to verify the consistency of intervention effects on pseudo-respondents.

Women in the action group who were not classified in the PM stage exhibited less evolution post-intervention than the others, as expected (see Additional file 1). The intervention is less novel for individuals in action stages who are already more engaged in healthy behaviors. Therefore, the intervention for this group aimed to prevent relapse and maintain behaviors [2].

Regarding the evolution of individuals who did not participate in the intervention, the usual care group (i.e., non-PM) exhibited increased lipid consumption. This result is concordant with global surveys showing increased fat intake among the general population [24], which reinforces the need and importance for interventions.

The main strength of this study is the validation of the algorithm used to classify individuals in the SOC for fat consumption. Furthermore, the interventions were based on the four pillars of the TTM. The cognitive and behavioral processes of change were strategically selected to promote motivation and progress through the SOC.

Despite this study’s strengths, some challenges and limitations are also present. The implementation of interventions for the pre-action and action subgroups may have limited the effects, because broad approaches that addressed all stages within in each group were required. However, 54 workshops were still held for these two groups. It is questionable whether workshops specific to the five SOC would be feasible in the context of health services.

It is difficult to compare this study with others because few intervention studies have evaluated the effectiveness of reclassifying individuals in the PM stage. Furthermore, the small sample size of the present study precluded identifying the statistical significance of other positive changes in the participants.

Future TTM-based interventions should assess the others pillars in changing eating behaviors (such as improved self-efficacy and reduced perceived barriers), which would expand knowledge about the cognitive and emotional dimensions of individuals. Evaluating these parameters will be important for advancing the understanding of the ability of interventions to positively affect individuals.

The present results regarding the PM stage must be verified by additional studies, which should also address the assumptions and questions raised herein. There was a high proportion of participants in the PM stage and a low proportion of participants in the non-reflective stages, and the individuals in the PM stage evolved distinctly despite participating in a nutritional intervention that was not tailored to their specific behaviors.

## Conclusion

Women attending public health promotion services may misperceive their lipid intake and not recognize the related dietary habits to a greater extent, especially with a high lipid intake. However, women in the PM stage who participated in TTM-based interventions for dietary fat consumption exhibited distinct positive changes compared with the other participants, indicating the importance of considering this classification in the evaluation of TTM-based interventions. Regardless, there is a paucity of research assessing the existence of the PM stage.

## Additional file

Additional file 1: Provides the results of the additional descriptive and exploratory analysis of the pre- and post-intervention measurements according to the PM classification, between the pre-action and action groups. (DOC 39 kb)

## Abbreviations

24DRs: 24-h dietary recalls
BMI: Body mass index
PM: Pseudo-maintenance
SOC: Stages of change
TM-IG: Transtheoretical Model intervention group
TTM: Transtheoretical Model
UCG: Usual care group
WHO: World Health Organization
