# Supplementary material for: Mistaken perception of lipid intake and its effects: a randomized trial
Source: BMC Nutr. 2017 Sep 19;3:77. doi: 10.1186/s40795-017-0193-8 (PMC7050849; doi:10.1186/s40795-017-0193-8)
Supplement: Supplementary file 1 — Provides the results of the additional descriptive and exploratory analysis of the pre- and post-intervention measurements according to the PM classification, between the pre-action and action groups. (DOC 39 kb) [file 40795_2017_193_MOESM1_ESM.doc]

**Descriptive statistics on pre- and post-intervention measurements according to the classification of pseudo-maintenance and pre-action and action group**

| **Transtheoretical Model-intervention group (n=40)** | | | | | | |
| --- | --- | --- | --- | --- | --- | --- |
| **Non–pseudo-maintenance group (n=21)** | | | | | | |
| **Characteristics** | **Pre-action Group (n=9)** | | | **Action Group (n=12)** | | |
|  | **Pre-intervention** | **Post-intervention** |  | **Pre-intervention** | **Post-intervention** |  |
| Calories (Kcal) | 1790,6±625,7 | 1462,4±516,4 |  | 1656,2±414,9 | 1523,4±277,9 |  |
| Weight (kg) | 68,5±9,0 | 67,1±8,4 |  | 67,7±12,8 | 67,5±13,3 |  |
| Body Mass Index (Kg/m^2^) | 28,7±2,2 | 27,9±1,9 |  | 28,3±4,8 | 28,3±5,0 |  |
| **Pseudo-maintenance group (n=19)** | | | | | | |
| **Characteristics** | **Pre-action Group (n=11)** | | | **Action Group (n=8)** | | |
|  | **Pre-intervention** | **Post-intervention** |  | **Pre-intervention** | **Post-intervention** |  |
| Calories (Kcal) | 1905,2±572,1 | 1696,5±336,9 |  | 2265,7  (1504,5;2846,1) | 1679,7  (1253,6;2073,1) |  |
| Weight (kg) | 66,7±11,1 | 65,5±11,0 |  | 71,3±13,7 | 70,0±13,3 |  |
| Body Mass Index (Kg/m^2^) | 26,9±3,3 | 26,4±3,3 |  | 28,5±5,4 | 28,0±5,2 |  |
